# Supplementary material for: The Interaction of Human and Epstein–Barr Virus miRNAs with Multiple Sclerosis Risk Loci
Source: Int J Mol Sci. 2021 Mar 13;22(6):2927. doi: 10.3390/ijms22062927 (PMC8000127; doi:10.3390/ijms22062927)
Supplement: Supplementary file 1 [file ijms-22-02927-s001.zip › Supplementary_Figures.docx]

**Supplementary Figures:**

**Figure S1:** Pipeline A. we used this workflow to identify MS risk miRNAs, obtain EBV miRNAs, and predict cellular and EBV miRNAs targets.

**Figure S2:** Pipeline B. The workflow for identifying MS risk SNPs or SNPs in LD with them which are located within or close to the putative EBV/cellular miRNAs target sites.

**Figure S3:** The list of genes which were targeted by MS risk miRNAs. The prediction algorithm was used for identifying the targeted genes illustrated by yellow, light green and dark green for TargetScan, miRmap and both, respectively. The asterisk indicates the MS risk genes.

**Figure S4:** The putative cellular miRNAs target sites which were located nearby the MS risk SNPs and SNPs in LD with them.

**Figure S1:** Pipeline A. we used this workflow to identify MS risk miRNAs, obtain EBV miRNAs, and predict cellular and EBV miRNAs targets.

**
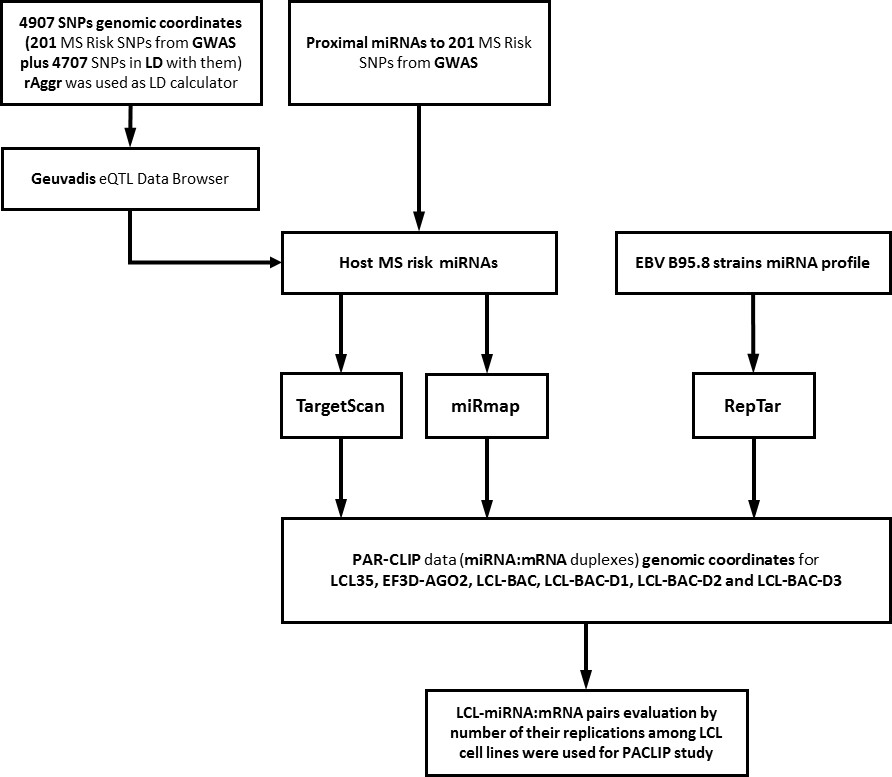
**

**Figure S2:** Pipeline B. The workflow for identifying MS risk SNPs or SNPs in LD with them which are located within or close to the putative EBV/host miRNAs target sites.


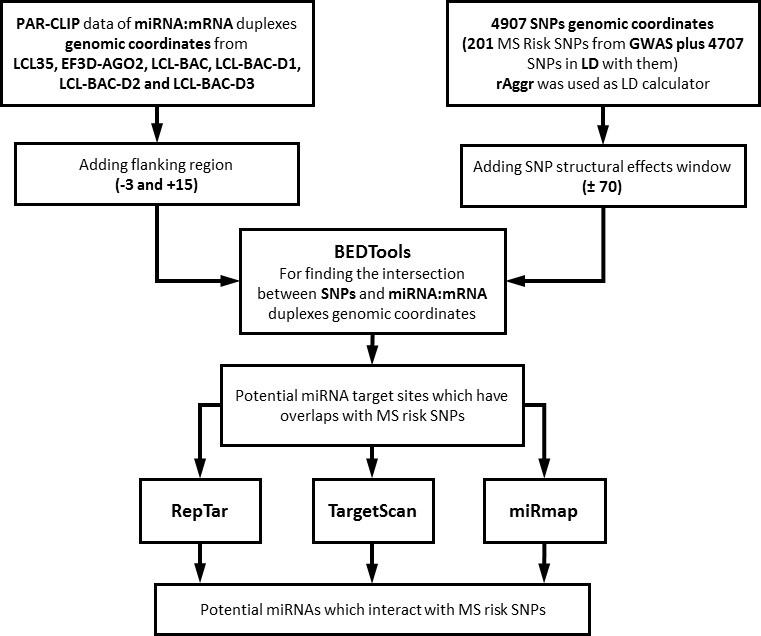


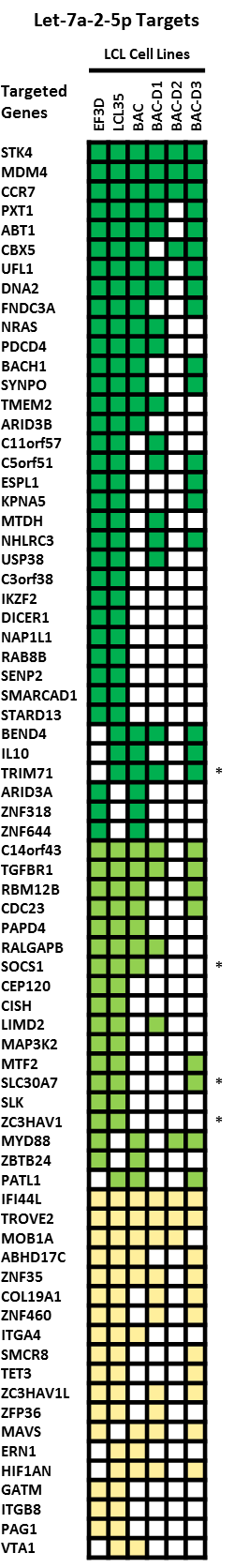

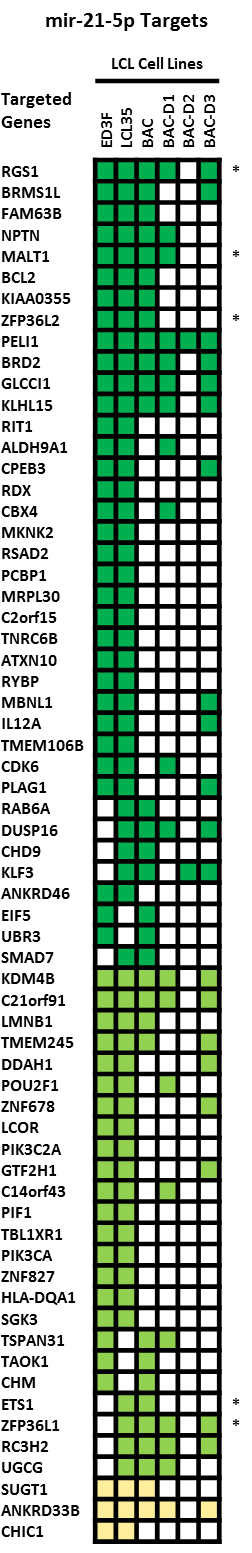

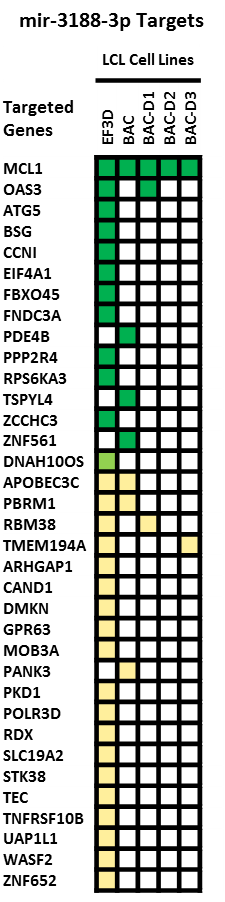

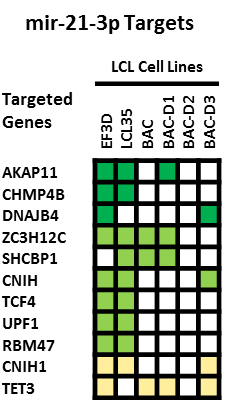


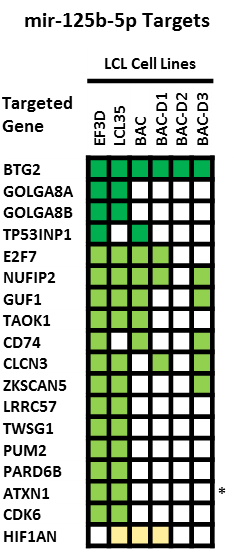


**Figure S3:** The list of genes which were targeted by MS risk miRNAs. Yellow, light green and Dark green shows the interactions predicted by TargetScan, miRmap and both, respectively. Star shows the MS risk genes.The asterisk indicates the MS risk genes.

**
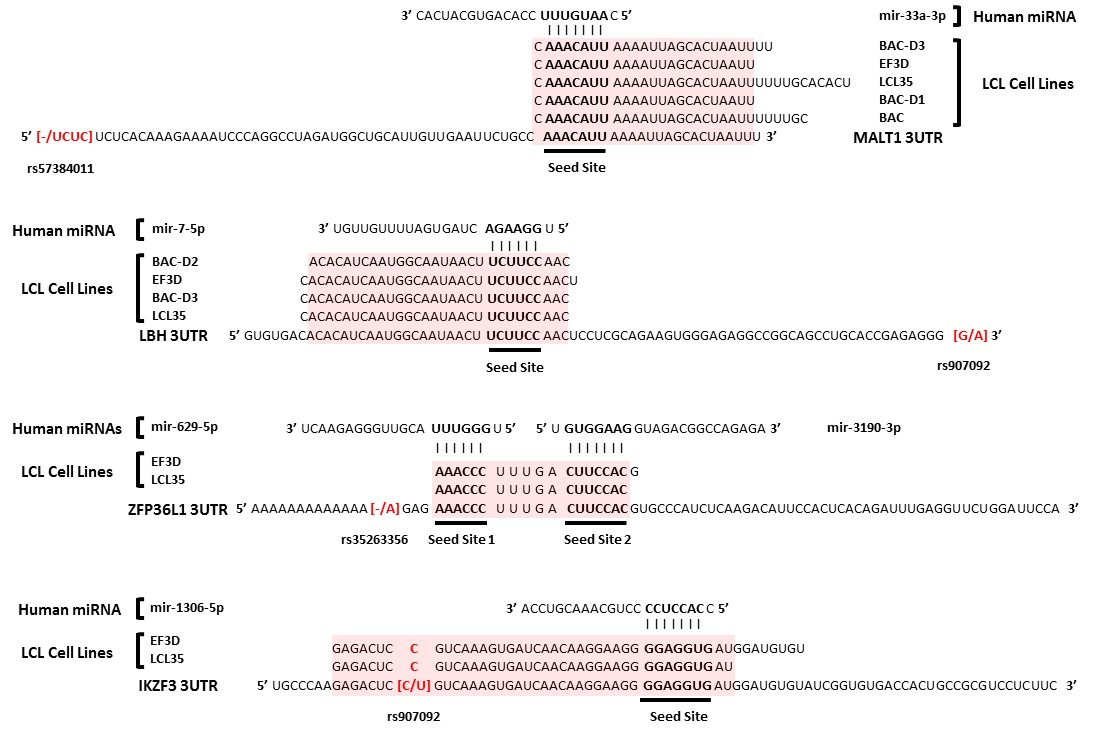
**

**Figure S4:** The putative cellular miRNAs target sites which were located nearby the MS risk SNPs and SNPs in LD with them.
