# Supplementary material for: The Interaction of Human and Epstein–Barr Virus miRNAs with Multiple Sclerosis Risk Loci
Source: Int J Mol Sci. 2021 Mar 13;22(6):2927. doi: 10.3390/ijms22062927 (PMC8000127; doi:10.3390/ijms22062927)
Supplement: Supplementary file 1 [file ijms-22-02927-s001.zip › Supplementary_Table_Legends.docx]

**Supplementary Tables:**

**Table S1:** List of MS risk miRNAs.

**Table S2:** eQTL effects of proximal MS risk SNP and the SNPs in LD with them on hsa-miR-3188-3p.

**Table S3:** Differentially expressed miRNAs in LCLs (n=5) versus B cells (n=5) using small RNA-seq.

**Table S4:** The genotype effect of proximal MS risk SNPs to MS risk miRNAs on the correlation between expression of MS risk miRNAs and their targeted genes in in the Euro LCL cohort (n=373) of Geuvadis study.

**Table S5:** Correlation between expression of MS risk miRNAs and their targeted genes in LCLs (n=5) and B cells (n=5).

**Table S6:** The conditional genotype effect of MS risk SNPs on the correlation between expression of MS risk miRNAs and their targeted MS risk genes in the Euro LCL cohort (n=373) of Geuvadis study.

**Table S7:** The genomic coordinate and feature of EBV miRNAs binding sites on MS risk genes.

**Table S8:** The genotype effect of proximal MS risk SNPs to MS risk genes on the correlation between expression of EBV miRNAs and their targeted MS risk genes in the Euro LCL cohort (n=373) of Geuvadis study.

**Table S9:** SNPs in LD with MS risk SNPs which are located within or adjacent to predicted miRNAs' target sites.

**Table S10:** Correlation between expression of MS risk genes which putative host miRNA target sites are located close to SNPs in LD with actual MS risk SNP.

**Table S11:** The genotype effect of proximal MS risk SNPs and SNPs in LD with them to MS risk genes on the correlation between expression of cellular miRNAs and their targeted MS risk genes in in the Euro LCL cohort (n=373) of Geuvadis study.
